# A random forest dynamic threshold imputation method for handling missing data in cognitive diagnosis assessments

**DOI:** 10.3389/fpsyg.2025.1487111

**Published:** 2025-08-05

**Authors:** Xiaofeng You, Jianqin Yang, Xinai Xu

**Affiliations:** School of Mathematics and Information Science, Nanchang Normal University, Nanchang, China

**Keywords:** missing data, cognitive diagnosis assessment, random forest threshold imputation, machine learning, dynamic thresholds

## Abstract

The handling of missing data in cognitive diagnostic assessment is an important issue. The Random Forest Threshold Imputation (RFTI) method proposed by You et al. in 2023 is specifically designed for cognitive diagnostic models (CDMs) and built on the random forest imputation. However, in RFTI, the threshold for determining imputed values to be 0 is fixed at 0.5, which may result in uncertainty in this imputation. To address this issue, we proposed an improved method, Random Forest Dynamic Threshold Imputation (RFDTI), which possess two dynamic thresholds for dichotomous imputed values. A simulation study showed that the classification of attribute profiles when using RFDTI to impute missing data was always better than the four commonly used traditional methods (i.e., person mean imputation, two-way imputation, expectation–maximization algorithm, and multiple imputation). Compared with RFTI, RFDTI was slightly better for MAR or MCAR data, but slightly worse for MNAR or MIXED data, especially with a larger missingness proportion. An empirical example with MNAR data demonstrates the applicability of RFDTI, which performed similarly as RFTI and much better than the other four traditional methods. An R package is provided to facilitate the application of the proposed method.

## Introduction

With the development of educational research, there is more demand for personalized feedback to enhance students’ learning in a targeted manner (Chatzopoulou and Economides, 2010; Hattie and Timperley, 2007; Parsons et al., 2018; Wu and Chang, 2023). To address this issue, Cognitive Diagnosis Models (CDMs) provide a useful psychometric framework that can finely classify students into different attribute profiles according to their responses on the test items (de la Torre and Minchen, 2014; Ketterlin-Geller and Yovanoff, 2009; Sia and Lim, 2018; You et al., 2019). However, the presence of missing responses is usually inevitable in such setting. For example, with the rise of personalized learning and hierarchical teaching in recent years, students often respond to only a portion of the items that match their ability instead of completing the entire test. Also, in large-scale assessments, the balanced-incomplete-block (BIB) design is typically used, in which examinees are administrated only a subset of items (Dai et al., 2018). In such cases, the data may be missing completely at random (MCAR), missing at random (MAR), missing not at random (MNAR), or even a mixture of two or three of those missingness mechanisms (e.g., Liu and Loken, 2025). Therefore, how to obtain accurate classifications of examinees’ attribute mastery status in diverse missing data scenarios is a crucial issue (Dai, 2017).

However, most commonly-used methods for handling missing data in the field of educational and psychological measurement have obvious limitations, and few methods are specifically designed for CDM applications. First, the existing methods (such as case deletion methods, regression imputation, likelihood-based estimation) often make assumptions about the missingness mechanism, whereas the mechanism is often unknown and even untestable and two or more mechanisms may be mixed in practice (Dai, 2017; de Ayala et al., 2001). Next, it is difficult for these methods to deal with a high missing data proportion (e.g., more than 30%), which however is common in practice due to test designs. Furthermore, whether the existing conclusions on these methods are applicable to CDMs needs further investigation. Current discussions about these methods are mostly based on Item Response Theory (IRT) models (Holman and Glas, 2005; Robitzsch, 2021), while some commonly-recommended methods have been found to perform quite differently in different research contexts (Dai, 2017; Newman, 2003; Song et al., 2022). For example, in Dai (2017)’s and Song et al. (2022)’s research based on cognitive diagnosis, the results of some popular methods, such as maximum likelihood (ML) and multiple imputation (MI), did not show obvious differences for MAR and MNAR data, which however should be expected in IRT contexts. For clarity, these commonly-used methods (such as case deletion methods, mean imputation, ML estimation, and MI) are collectively referred to as traditional methods in this article, as opposed to the machine-learning-related approaches that will be introduced later.

Since the early 2000s, a new framework for imputing missing values through machine learning algorithms has emerged, in which a machine learning model is trained based on the data samples with observed values for specific variables and then predict the missing values (Liu and Gopalakrishnan, 2017). This type of approach is gaining popularity due to their applicability and effectiveness in handling large datasets (Thomas and Rajabi, 2021). According to Thomas and Rajabi’s (2021) review of machine learning-based missing data imputation techniques during 2010 ~ 2020, clustering, instance-based (e.g., k-nearest neighbor or KNN), and ensemble (e.g., random forest) techniques are the most popular algorithms applied for data imputation. These techniques are nonparametric algorithms that make no parametric assumptions about the relationship between variables. Thus, when employed in data imputation, they do not require strong assumptions about the missingness mechanism, and have been found to always perform well (e.g., Kokla et al., 2019; Richman et al., 2009; Suresh et al., 2019). Due to these advantages of such new approaches, how to incorporate them into the measurement models is getting more and more attention.

In response to the issue of missing data in the implementation of CDMs and the increasing popularity of machine learning imputation methods, You et al. (2023) proposed a Random Forest Threshold Imputation (RFTI) method, which is an adaption of the Random Forest Imputation (RFI; Stekhoven and Büehlmann, 2012). Simply put, when imputing missing values for items scored as 0–1, for each unobserved value, RFI first predicts a probability value within [0,1] based on the random forest algorithms, which indicates the probability of the missing value taking the value of 1. In other words, the closer the probability value is to 1, the unobserved value is more likely to be 1; otherwise, the value is more likely to be 0. The probability value is then converted to a dichotomous value of 0 or 1 using a fixed threshold (e.g., 0.5). In reality, however, as the probability value approaches 0.5, the missing value is more likely to be incorrectly imputed, regardless of whether the imputed value is 0 or 1, due to the high uncertainty in the model prediction. Therefore, You et al. (2023) proposed to leave the missing value with high uncertainty still missing and only impute those with predicted probability values close to 0 or 1. So, RFTI utilizes two thresholds for the dichotomous imputed value, of which the lower one is fixed at 0.5 and the upper one is dynamic and determined by an adapted person fit index in CDMs. Therefore, RFTI is designed for CDMs based on a machine learning imputation algorithm and has been found to be superior in the recovery of examinees’ mastery profiles than RFI and the expectation–maximization (EM) algorithm (a general method to perform ML estimation on incomplete data), especially for MNAR and MIXED data and a large missingness proportion (You et al., 2023).

The idea of setting a dynamic threshold is worthwhile. However, it can be noticed that the lower threshold is still fixed at 0.5 in RFTI. Although You et al. (2023) mention that it is reasonable to consider the unobserved responses as wrong (i.e., replace them with 0) in the educational assessment if the predicted probability is 0.5 or below, there is no substantial evidence to support this claim. Replacing missing values with 0 for which the predicted probability is less than but close to 0.5 is still subject to high uncertainty. It remains unknown that whether this will influence the imputation accuracy and hence the classification accuracy of examinees’ attribute patterns. Besides, the simulation conditions in You et al. (2023) are limited in that they only varied the missing data mechanisms and missingness proportions without taking into account other factors in the actual cognitive diagnostic assessments, and only three methods, including EM, RFI and RFTI, were compared.

Therefore, in this study, we proposed an improved version of RFTI, in which both upper and lower thresholds for converting the predicted probability values to dichotomous values are dynamic to fully account for imputation uncertainty, and we call the new method as Random Forest Dynamic Threshold Imputation (RFDTI) method. In the following sections, we first briefly introduce the four missing data mechanisms and several traditional methods that are commonly used in educational and psychological measurements, especially cognitive diagnosis contexts. Then, we describe the principles and ideas of the RFTI method in detail and lead to the improved version, the RFDTI method, on this basis. Afterward, we show a Monte Carlo simulation study, in which we systematically investigated the performance of the proposed RFDTI method under different conditions from the perspective of the classification accuracy of CDMs and compared it with RFTI as well as several commonly used traditional missing data handling methods. An empirical example is also provided to illustrate the applicability of the proposed method in practice. Finally, we conclude the paper with a discussion.

### Missing data mechanisms and traditional handling methods

Missing data and their treatment would substantially affect the analysis results based on such data (Cheema, 2014; Little and Rubin, 2002; Tabachnick and Fidell, 1989). Therefore, appropriate techniques for handling missing data should be adopted, and the method selection is usually based on the mechanism and proportion of missingness, as well as the purpose and model of data analysis (Little and Rubin, 2002; Tabachnick and Fidell, 1989).

Little and Rubin (2002) defined three types of missing data mechanisms, i.e., MCAR, MAR, and MNAR. Under the MCAR mechanism, the probability of missingness is unrelated to both observed and unobserved data, so the missing values can be completely ignored in the analysis. When data are MAR, missing data in a particular variable are related to some measured variables in the dataset but are unrelated to that variable itself. For example, the missingness is conditional on other measurable characteristics of the examinee but not on the item score in which missingness occurs. The MNAR mechanism refers to the situation in which the missingness on a variable is partly or completely related to the unobserved values in that variable. For example, the missingness proportion of a difficult item is high, while that proportion of an easy item is low. Therefore, MNAR is considered nonignorable. In addition to the three mechanisms mentioned above, there is a MIXED type of missing data mechanism that was used in de Ayala et al. (2001) and Dai (2017). Based on an empirical dataset, de Ayala et al. (2001) found that item responses (correct, incorrect, or an omitted response) of examinees were related to both the person’s ability and the items. Because a test-taker may omit an item for different reasons in practice and these factors cannot be explicitly measured currently, we also include the MIXED mechanism in this study.

Based on previous studies (e.g., Dai and Svetina Valdivia, 2022; Song et al., 2022), here we review four categories of the traditional methods for handling missing data, which may be commonly used in cognitive diagnosis contexts: case deletion, single imputation, ML estimation, and MI (Gemici et al., 2012; Schafer and Graham, 2002). Case deletion methods, including listwise and pairwise deletion, are popular and easy to implement, but often result in a large amount of information loss, thereby decreasing statistical power. Commonly-used single imputation methods include person mean imputation (PM) and two-way imputation (TW). PM imputes each missing value using the corresponding respondent’s mean score across all available items. TW method further takes into account information from the item mean and the grand mean in addition to the person mean. These two methods are also easy to implement and are robust in dealing with missing values in multidimensional data (e.g., Bernaards and Sijtsma, 2000). As for the ML estimation, a general method to perform it on incomplete data is EM algorithm, which iterates between an expectation step and a maximization step. In the expectation step, missing values are filled in using the expectation based on the current estimates of unknown parameters, whereas in the maximization step, the parameters are re-estimated from the observed and filled data. Strictly speaking, EM is also a single imputation method, but it is stochastic, unlike the deterministic PM and TW. Another ML method is the direct maximum likelihood (also known as full information maximum likelihood), which maximizes the likelihood function directly based on parameters from a specified distribution, rather than first imputing missing values. Therefore, this method is sometimes labeled as the “available cases” approach in some software.

MI, as a flexible alternative to likelihood methods, is not a specific imputation method but rather a multi-step imputation framework. In MI, each missing value is substituted by *m* > 1 simulated values, resulting in *m* imputed datasets. Each of the *m* datasets is then analyzed using the desired statistical analysis method in the same manner. Finally, the results are pooled by simple arithmetic to produce overall estimates and standard errors (Schafer and Graham, 2002). Theoretically speaking, any stochastic imputation method (such as EM and regression-based methods) can be used with MI. In general, likelihood methods and MI, both considered model-based methods, have been suggested as the optimal approaches for handling missing data in many situations (Finch, 2008; Schafer and Graham, 2002; van Buuren, 2018; Wothke, 2000). Nevertheless, each method for treating missing data has its own features and assumption, and no one method can consistently outperform the others under different circumstances (Finch, 2008).

Regardless of the specific limitations of each method, all these traditional missing data handling methods are subject to the following issues. First, these methods require (strong) statistical assumptions, including the assumption that the missingness mechanism is MCAR or MAR, which may not be satisfied in practice. However, traditional methods often perform poorly under the MNAR mechanism, and what is worse is that the MNAR mechanism is difficult to test in advance. Second, most methods can provide desirable results only when the missingness proportion is not high. In previous simulation studies on missing data handling methods, the specified missing data proportions ranged from 2% (de Ayala et al., 2001) to 50% (Glas and Pimentel, 2008), most of which were between 5 and 30% (Finch, 2008). These methods do not work well when the proportion exceeds 20%, and a large bias may occur in the estimation when the proportion reaches above 30%. Third, although a variety of approaches has been developed to deal with the problem of missing responses in educational measurement, most of them are within the IRT framework (Dai and Svetina Valdivia, 2022). For other complex measurement models, such as CDMs, there are few missing data handling methods that take into account the characteristics of the model itself.

## Random forest threshold imputation

The rise of machine learning provides a new paradigm for imputing missing values. Machine learning models can be trained based on the observed data and then used to predict missing values. Due to the underlying machine learning algorithms, this type of imputation method is often not as dependent on assumptions of missingness mechanisms as the traditional methods mentioned above and often performs better (e.g., Kokla et al., 2019; Richman et al., 2009; Suresh et al., 2019).

You et al. (2023) incorporated the Random Forest Imputation (RFI; Stekhoven and Büehlmann, 2012), a flexible and effective machine learning imputation method, with the features of CDMs to develop a Random Forest Threshold Imputation (RFTI) method. It is specially designed for CDMs and possesses the advantages of machine learning imputation methods.

### Basic procedure

The key idea of RFTI is building on the random forest imputation, that is, it allows some missing values with low certainty of imputation to remain missing, which is realized by setting two thresholds:


(1) $$Y_{\mathrm{ij}}=\{\begin{array}{c} 1p_{\mathrm{ij}}\geq \tau \\ \mathrm{NA}\;0.5<p_{\mathrm{ij}}<\tau \\ 0p_{\mathrm{ij}}\leq 0.5 \end{array}$$

in which $Y_{\mathrm{ij}}$ denotes the imputed response of examinee *i* on item *j*, $p_{\mathrm{ij}}$ is the imputed probability for examinee *i* on item *j*, *NA* represents missingness, τ is the dynamic upper threshold and 0.5 is the fixed lower threshold. For the same dataset, τ will be substituted for a range of possible values $\left\{\tau^{\left(1\right)},\ldots ,\tau^{\left(T\right)}\right\}$ within a reasonable range of [0.5, 1) in evenly spaced increments (e.g., 0.01), and its final value will be the one yielding the best imputation effect, which is evaluated using an adapted person fit index in CDMs. Therefore, for each $\tau^{\left(t\right)},t=1,2,\ldots ,T,$ the following procedures of imputation and model fit will be repeated.

Suppose that there is an *N* × *M* data matrix Y , where *N* denotes the number of examinees and *M* is the number of variables (i.e., test items). Then it can be viewed as $Y=(Y_{1},Y_{2},\ldots ,Y_{M})$ , in which $Y_{m}$ is the collection of all examinees’ responses on the *m*th item ( m=1,2,…,M ). Let $Y_{s}\;(s\in 1,2,\ldots ,m)$ denote an arbitrary variable with missing data, $i_{\mathrm{mis}}^{\left(s\right)}\in \{1,2,\ldots ,N\}$ denote the examinees with missing values in $Y_{s}$ , and $i_{\mathrm{obs}}^{\left(s\right)}\in \{1,2,\ldots ,N\}$ denote the remaining examinees with observed values in $Y_{s}$ . Subsequently, the dataset can be divided into four parts: (1) $y_{\mathrm{obs}}^{\left(s\right)}$ , representing the observed values in variable $Y_{s}$ ; (2) $y_{\mathrm{mis}}^{\left(s\right)}$ , representing the missing values in $Y_{s}$ ; (3) $x_{\mathrm{obs}}^{\left(s\right)}$ , representing the data of examinees $i_{\mathrm{obs}}^{\left(s\right)}$ in all other (m−1) variables except $Y_{s}$ ; (4) $x_{\mathrm{mis}}^{\left(s\right)}$ , representing the data of examinees $i_{\mathrm{mis}}^{\left(s\right)}$ in all other (m−1) variables except $Y_{s}$ . The imputation procedure is an iterative process involving the following steps.

The first step is to use a traditional imputation method, such as the item mean imputation, to calculate the initial estimates of all the missing values. Then, sort all variables with missingness, $Y_{s}\;(s\in 1,2,\ldots ,m)$ , in the ascending order of the number of missing values. The imputed matrix is denoted by $Y_{\mathrm{old}}^{\mathrm{imp}}.$

The second step is to impute the missing values for each $Y_{s}$ through the random forest algorithm, in which converting probability values to dichotomous values using Equation 2. Specifically, to conduct imputation for the variable $Y_{s}$ , a random forest model is trained using $y_{\mathrm{obs}}^{\left(s\right)}$ as the response and $x_{\mathrm{obs}}^{\left(s\right)}$ as the predictors. Then, the fitted model is applied to predict the missing values $y_{\mathrm{mis}}^{\left(s\right)}$ using $x_{\mathrm{mis}}^{\left(s\right)}$ as input. Notice that at this time, the predicted probability values provided by the fitted model are converted to dichotomous values (0 or 1) based on the prespecified upper threshold $\tau^{\left(t\right)}$ and fixed lower threshold 0.5. This process is repeated for all variables with missing values. After completing the imputation for all $Y_{s}$ , the new imputed matrix obtained is denoted by $Y_{\mathrm{new}}^{\mathrm{imp}}$ and then compared to $Y_{\mathrm{old}}^{\mathrm{imp}}$ .

If the difference between the two imputed matrices does not meet the stopping criterion, the next iteration will be carried out. In the new iteration, $Y_{\mathrm{new}}^{\mathrm{imp}}$ in the previous iteration will be assigned to $Y_{\mathrm{old}}^{\mathrm{imp}}$ , and the second imputation step will be repeated to update $Y_{\mathrm{new}}^{\mathrm{imp}}$ . The stopping criterion is that the difference between $Y_{\mathrm{new}}^{\mathrm{imp}}$ and $Y_{\mathrm{old}}^{\mathrm{imp}}$ increases for the first time. For the set of M discrete variables, that difference is measured by Δ , which is calculated by Equation 2.


(2) $$\Delta =\frac{\sum_{j=1}^{M}\sum_{i=1}^{N}I_{Y_{\mathrm{new}}^{\mathrm{imp}}\neq Y_{\mathrm{old}}^{\mathrm{imp}}}}{\#\mathrm{NA}}$$

in which #NA is the number of all missing values in the matrix Y, and $I_{Y_{\mathrm{new}}^{\mathrm{imp}}\neq Y_{\mathrm{old}}^{\mathrm{imp}}}$ is an indicator variable that records whether the imputed value in Row *i,* Column *j* differs between two successive iterations. If that value differs, I=1 ; otherwise, I=0 . Therefore, the numerator in Equation 2 represents the number of imputed values that change between two iterations (Stekhoven and Büehlmann, 2012).

After obtaining a final imputed data matrix related to $\tau^{\left(t\right)}$ , a CDM selected by researchers is fitted to this data matrix, in which the EM algorithm is used for item parameter estimation and the maximum a posterior (MAP) method is used to estimate latent attribute patterns. The remaining missing values after imputation are simply ignored. Based on the estimated attribute patterns and Q-matrix expectations, an adapted person fit index is calculated for evaluating the imputation accuracy for each $\tau^{\left(t\right)}$ .

### Determination of the upper thresholds τ

The determination of the upper thresholds ( τ ) is a balance between the imputation proportion and imputation accuracy in actual situations. A higher τ will result in fewer but more accurate imputed values. On the other hand, the missingness proportion in the imputed dataset should be low enough (preferably less than 10, 10% ~ 15% sometimes acceptable) (e.g., Dai, 2017; Hair et al., 2010; Little and Rubin, 2002; Muthén et al., 2011), so that simply ignoring these remaining missing values in the subsequent analysis will not bring a substantial bias.

Logically, the imputed values with high certainty should not damage the overall fit between the data and the expectations of the CDM used in the analysis. The more errors in imputed values, the greater the deviation of the imputed data from the ideal response patterns. When the deviation is large enough, the imputation should be stopped. Out of this consideration, You et al. (2023) adapted the response conformity index (RCI) proposed by Cui and Li (2015), which is a person fit index in CDMs, to evaluate the deviation of the imputed data using a possible value $\tau^{\left(t\right)}$ of τ from the ideal response patterns based on the current estimated model.

The adapted index is calculated in two steps. In the first step, an $\mathrm{RCI}\_C_{i}$ is calculated for each examinee *i* as


(3) $$\mathrm{RCI}\_C_{i}=\frac{\sum_{j=1}^{m_{i}}|\ln{\left[-\frac{Y_{\mathrm{ij}}-P_{j}({\hat{\alpha }}_{i})}{I_{j}({\hat{\alpha }}_{i})-P_{j}({\hat{\alpha }}_{i})}\right]}^{Y_{\mathrm{ij}}+I_{j}({\hat{\alpha }}_{i})}|}{m_{i}}$$

in which $m_{i}$ ( $0<m_{i}\leq M$ ) is the number of nonmissing items for examinee *i* in the imputed data matrix with M items; $Y_{\mathrm{ij}}$ denotes the observed or imputed response of examinee *i* on item *j*; ${\hat{\alpha }}_{i}$ represents the estimated attribute profile of examinee *i* since the true profile is unknown in practice; $P_{j}({\hat{\alpha }}_{i})$ denotes the probability of a correct response to item *j* given $\alpha_{i}$ , $I_{j}({\hat{\alpha }}_{i})$ is the corresponding ideal response, and $I_{j}({\hat{\alpha }}_{i})=1\;$ only if examinee *i* masters all the attributes required by item *j*, otherwise, $I_{j}({\hat{\alpha }}_{i})=0$ . The Q matrix specifies the attributes required for each item. In the second step, the mean of $\mathrm{RCI}\_C_{i}$ is calculated across all examinees, that is,


(4) $$\bar{\mathrm{RCI}\_C}=\frac{\sum_{i=1}^{N}\mathrm{RCI}\_C_{i}}{N}$$

Therefore, for each $\tau^{\left(t\right)}$ , a value of $\bar{\mathrm{RCI}\_C}$ can be obtained. More accurate imputations will result in imputed response patterns that are more consistent with expectations, thereby generating a smaller $\bar{\mathrm{RCI}\_C}$ .

In { $\tau^{\left(1\right)},\ldots ,\tau^{\left(T\right)}$ }, the value resulting in the smallest $\bar{\mathrm{RCI}\_C}$ is selected as the optimal upper threshold, and the corresponding imputed data matrix is the final imputed result that will be used in the subsequent analysis. In practical application, it is sufficient to export the final imputed data matrix only.

## Random forest dynamic threshold imputation

RFTI is built on the random forest imputation algorithms while leveraging the characteristics of CDMs to dynamically determine the upper threshold to reduce the imputation errors. However, according to Equation 1, the lower threshold is still fixed at 0.5 in RFTI. That is, any missing value with a predicted probability not greater than 0.5 is replaced with 0. When thinking about the starting point of RFTI to reduce estimation uncertainty, this is puzzling. Replacing missing values with 0 for which the predicted probability approaches 0.5 is still subject to a high degree of uncertainty, and so carries the risk of imputation errors. Although You et al. (2023) mention that it is reasonable to consider the unobserved responses as wrong (i.e., replace them with 0) in the educational assessment if the predicted probability is 0.5 or below, there is no substantial evidence to support this claim in their study. It remains unknown that whether this will influence the imputation accuracy and hence the classification accuracy of examinees’ attribute patterns.

Therefore, in this study, we proposed an improved version of RFTI, i.e., Random Forest Dynamic Threshold Imputation (RFDTI). The key difference between them is that in RFDTI, both upper and lower thresholds ( $\tau_{u}$ and $\tau_{l}$ ) for converting the predicted probability values to dichotomous values are dynamic to fully account for imputation uncertainty. In other words, the following Equation 5 is used instead of Equation 1 when determine the imputed dichotomous values.


(5) $$Y_{\mathrm{ij}}=\{\begin{array}{c} 1p_{\mathrm{ij}}\geq \tau_{u}\\ \mathrm{NA}\tau_{l}<p_{\mathrm{ij}}<\tau_{u}\\ 0p_{\mathrm{ij}}\leq \tau_{l} \end{array}$$

in which $0<\tau_{l}<0.5$ and $0.5\leq \tau_{u}<1$ . The dynamic upper and lower thresholds will be simultaneously determined based on the adapted person fit index $\bar{\mathrm{RCI}\_C}$ , while other procedures are the same as those of RFTI. That is, for each combination of $\tau_{l}^{\left(t_{l}\right)}\;(t=1,2,\ldots ,T_{l})$ and $\tau_{u}^{\left(t_{u}\right)}\;(t_{u}=1,2,\ldots ,T_{u})$ , an imputed data matrix can be obtained according to the predicted probability of missing values and Equation 5, for which an $\bar{\mathrm{RCI}\_C}\;$ can be calculated according to Equations 3, 4. The combination of $\tau_{l}$ and $\tau_{u}$ values corresponding to the smallest $\bar{\mathrm{RCI}\_C}$ is selected as the optimal thresholds, and the corresponding imputed data matrix will be used in the subsequent analysis.

To facilitate the application of the proposed RFDTI, we developed the corresponding R package *missForestCDA*, which can be downloaded from https://jianlingsoft.oss-cn-beijing.aliyuncs.com/missForestCDA.rar. This package requires preinstallation of the R package *missForest* (Stekhoven, 2013) for implementing the random forest imputation and the R package *CDM* (George et al., 2016; Robitzsch et al., 2017) for the estimation of CDMs. After installing and loading the *missForestCDA* package, the incomplete dataset can be imputed by simply handing it over to the main function of this package:


missForestCDA(missData,Q,missN,stepV=0.05)

in which *missData* is the input incomplete dataset, *Q* is the Q matrix that needs to be specified by researchers, *missN* is the number of persons with missing responses, and *stepV* is the increment δ used to generate a sequence of possible values for $\tau_{l}$ and $\tau_{u}$ , which is set at 0.05 by default. This function will directly return the final imputed dataset.

In this study, we conducted a Monte Carlo simulation study and an empirical study to investigated the performance of the proposed RFDTI method under different conditions and compared it with RFTI as well as several commonly used traditional missing data handling methods. According to Equations 3, 4, the adapted person fit statistic $\bar{\mathrm{RCI}\_C}$ can be obtained for any CDMs with explicitly defined item response function $P_{j}(\alpha_{i})$ . In this study, for the purpose of illustration, the generalized Deterministic Inputs, Noisy and Gate (DINA) model (de la Torre, 2011) is used as an example.

Note that in You et al. (2023)’s study, the superiority of RFTI over RFI and EM was mainly in the classification of attribute profiles, whereas the estimation of item parameters using RFTI was inferior to that of EM. This may be because the training and prediction of the random forest model underlying RFTI are mainly based on the characteristics of individual response patterns across items, while information about responses from different examinees to the same item is rarely used. Based on this, we focus on the classification of attributes’ mastery status in this study, leaving aside the item parameter estimation temporarily.

## Simulation study

A Monte Carlo simulation study was conducted to investigate the imputation effect of the proposed RFDTI method under different missingness conditions and its relative performance compared with the RFTI as well as four commonly used imputation methods in educational assessments.

### Design

A total of 4 × 5 × 3 × 6 = 360 conditions were created by manipulating four factors, including the missing data mechanism (MIXED, MNAR, MAR, and MCAR), missingness proportion (10, 20, 30, 40, and 50%), sample size (*N* = 500, 1,000 and 2000) and the number of attributes (*K* = 3, 4, 5, 6, 7, 8). The missing data proportion and the number of attributes were chosen according to common settings in related studies. Specifically, the missing rate reported in the educational measurement literature, as mentioned above, was between 2 and 50%, and most existing CDM studies used three to eight attributes (Dai, 2017). In addition, a sample size of 1,000 was widely used (Dai, 2017) and was considered sufficient for the DINA model to obtain an accurate parameter estimation (de la Torre et al., 2010). Therefore, we considered three levels of sample size centered at 1000. Each simulation condition was replicated 100 times. Each generated dataset was imputed using six approaches, including RFTI, RFDTI, and four frequently used methods in educational assessments, including PM, TW, EM, and MI.

Other specifications reflected the common settings in simulations and empirical studies of CDMs reported in previous literature. According to the review of CDM studies by Dai (2017), the number of items was mostly between 20 and 40, so a test length of 30 items was used here. For simplicity, we assumed that attributes were independent of each other. The Q-matrix reflecting the mapping relationship between attributes and items was randomly generated. Specifically, q-entries in the Q-matrix were randomly drawn from the uniform distribution U(0,1) and then dichotomized by the cut-off point of 0.5. Therefore, each item might measure one or more attributes.

### Data generation

Data generation was implemented in R language and involved two steps: generating the complete datasets and then generating the missing data.

#### Complete data generation

First, the DINA model was used to simulate the complete dichotomous responses under each condition. In the DINA model, the item response probability is written as:


(6) $$\begin{array}{l} P_{j}(\alpha_{i})=P(X_{\mathrm{ij}}=1|\alpha_{i})\\ =g_{j}^{1-\eta_{\mathrm{ij}}}{\left(1-s_{j}\right)}^{\eta_{\mathrm{ij}}}=\{\begin{array}{c} g_{j}\text{if}\;\eta_{\mathrm{ij}}=0\\ 1-s_{j}\text{if}\;\eta_{\mathrm{ij}}=1 \end{array} \end{array}$$

in which $X_{\mathrm{ij}}$ is the response of examinee *i* to item *j*, $\alpha_{i}=(\alpha_{i1},\ldots ,\alpha_{\mathrm{iK}})$ is the examinee’s attribute profile, $g_{j}$ is the guessing parameter of item *j*, $s_{j}$ is the slipping parameter of item *j*, $\eta_{\mathrm{ij}}=\prod_{k=1}^{K}\alpha_{\mathrm{ik}}^{q_{\mathrm{jk}}}$ is the ideal response of examinee *i* to item *j*, and $q_{\mathrm{jk}}$ is the element in the Q matrix indicating whether attribute *k* is required for a correct response to item *j*.

Following the literature (e.g., Cui et al., 2012; Dai and Svetina Valdivia, 2022), examinees’ attribute profiles ( $\alpha_{i}$ ) were generated from a dichotomized multivariate normal distribution $\mathrm{MVN}(0_{K},\Sigma )$ . Specifically, $0_{K}$ is a 1×K vector of zeros with *K* being the number of attributes measured by the test, Σ is a K×K covariance matrix with all diagonal elements being 1 and all off-diagonal elements being 0.5, as shown in Equation 7:


(7) $$\Sigma =[\begin{array}{ccc} 1 & \ldots & 0.5\\ \vdots & \ddots & \vdots \\ 0.5 & \ldots & 1 \end{array}]$$

and the cut point for each attribute was set to zero. Item parameters in the DINA model, including the slipping parameter *s* and the guessing parameter *g*, were drawn from the uniform distribution [0.05, 0.25]. Then, according to Equation 6, the probability of a correct response of examinee *i* to item *j*, $P_{j}(\alpha_{i})$ , was calculated and compared to a uniform random number [0, 1]. If $P_{j}(\alpha_{i})$ was not less than the random number, the response $X_{\mathrm{ij}}$ was coded as 1 for correct; otherwise, $X_{\mathrm{ij}}=0$ .

#### Missing data generation

Considering that the trained models in RFTI and RFDTI methods are based on examinees with observed data on the target variable, the training accuracy can be improved if there are some examinees with complete response data. Therefore, we randomly selected 80% of the sample (e.g., 800 out of 1,000 simulated examinees) to generate missing values, leaving a small number of examinees with complete data. Note, however, that the application of the two methods do not require some examinees in the sample have complete data.

MCAR missing samples were simulated by randomly removing a specified percentage of responses from the complete dataset. This was achieved by comparing the specified overall missingness proportion (e.g., 30%) with a uniform random number [0, 1] generated for each response. If the random number was greater than or equal to the proportion, the corresponding response was removed as missing.

Generation of missing responses of MAR followed the methods outlined in de Ayala et al. (2001), Peugh and Enders (2004), and Finch (2008). Based on the complete dataset, the number-correct score was calculated for each examinee on all but the target item as an ability-proxy variable. Examinees were divided into seven fractiles based on the 5th, 15th, 30th, 70th, 85th, and 95th percentiles of their normalized scores on the proxy variable. Examinees of each fractile were assigned a missingness probability that was inversely related to their scores (see Table 1), while the average missing rate across fractiles was kept at the desired level. Uniform random numbers [0, 1] were used to select responses for deletion according to the missing rate of each fractile.

**Table 1:** MAR missing rate of each fractile.

Percentile	Missing rate (%)
0 ~ 5th	MR × 1.50
5 ~ 15th	MR × 1.35
15 ~ 30th	MR × 1.15
30 ~ 70th	MR × 1.00
70 ~ 85th	MR × 0.85
85 ~ 95th	MR × 0.65
95 ~ 100th	MR × 0.50

MR = the desired overall missing rate in each condition. Fractiles are represented by the percentiles of the normalized scores on the proxy variable.

Following the method outlined in Dai (2017), the MNAR data were generated by calculating the omission probability based on the responses in the complete dataset. Examinees with an incorrect response to an item were assigned a higher probability of omission than those who answered the item correctly. In addition, the missing rate on items increased with the item difficulty. The generation procedure is as follows. The number of omitted responses for each examinee was first calculated based on the desired overall missingness proportion. A probability factor ε was then specified for each examinee, with an initial value of 0. A uniform random number [0, 1] was compared with p+ε to determine whether to remove a response as an omission, in which p is the probability of a correct response. If the random number was larger, the response was removed. During the procedure, if the number of missing responses for an examinee was greater (or less) than the prespecified number, the value of ε would be increased (or decreased). For each examinee, the value of ε was constantly adjusted to regenerate the missing data until the number of omitted items was equal to the desired number.

To generate data of the MIXED mechanism, we first adopted the same procedure as generating the MAR data, that is, dividing the sample into seven fractiles and calculating the missing rate of each fractile, in which examinees with higher scores had a lower missingness proportion. Subsequently, the number of missing responses for each examinee could be calculated according to the assigned fractile, and the omissions were generated through the procedure of generating the MNAR data mentioned above.

### Analysis

All missing data were imputed using the corresponding R packages. Specifically, PM, TW, and EM were conducted using the *TestDataImputation* package (Dai et al., 2021). MI was carried out using function mice() in *mice* package (van Buuren et al., 2021), in which the specific imputation method used was logistic regression imputation and 20 imputed datasets were created for each incomplete dataset (Graham et al., 2007). RFTI and RFDTI were implemented with the *missForestDINA* and *missForestCDA* packages, respectively.

After the imputation, the DINA model was fit to the data using the R package *CDM* (Robitzsch et al., 2017). The estimation of examinees’ attribute profiles, as the focus of this study, was then evaluated across all 100 replications in each condition. Note that when using MI for imputation, since attribute profiles were dichotomous data, their estimation accuracy results (rather than estimates) were pooled by averaging the corresponding results across multiple imputations.

### Evaluation criteria

As this study focuses on the classification of attribute mastery status, we adopted two relevant criteria to evaluate the performance of each imputation method: the pattern-wise classification accuracy (PCA) and the attribute-wise classification accuracy (ACA).


(8) $$\mathrm{PCA}=\sum_{r=1}^{R}\sum_{i=1}^{N}I[{\hat{\alpha }}_{i}=\alpha_{i}]/(R\times N)$$

(9) $$\mathrm{ACA}=\sum_{r=1}^{R}\sum_{k=1}^{K}\sum_{i=1}^{N}I[{\hat{\alpha }}_{\mathrm{ik}}=\alpha_{\mathrm{ik}}]/(R\times K\times N)$$

where ${\hat{\alpha }}_{i}=({\hat{\alpha }}_{i1},\ldots ,{\hat{\alpha }}_{\mathrm{iK}})$ and $\alpha_{i}=(\alpha_{i1},\ldots ,\alpha_{\mathrm{iK}})$ are the estimated and true attribute patterns for examinee *I*, respectively, and I[·] is an indicator function that takes the value of 1 or 0 depending on whether the condition in brackets is met; *R* is the number of successfully converged replications in each condition; *N* is the sample size; and *K* is the number of attributes. PCA measures the average classification accuracy of examinees’ attribute patterns, and ACA measures the average classification accuracy of the attributes. A larger value of PCA or ACA indicates a more accurate classification of the attribute mastery status.

## Results

### Missing rate of data imputed by RFDTI

Considering that RFDTI may not impute all missing values and a low proportion of missing data may be retained and ignored in the following analysis, we first examined the remaining missing rates of the data imputed by RFDTI in different conditions. Due to the same issue faced by RFTI, we also provided the results from RFTI for comparison. Results showed that the missing rate of RFDTI imputed data was mainly affected by the missingness mechanism and proportion, while the sample size and the number of attributes had little effect. Therefore, the missing rates of RFDTI imputed data under different missingness mechanisms and proportions are listed in Table 2.

**Table 2:** Remaining missing rates of data imputed by RFDTI and RFTI under different missing mechanisms and proportions.

Missingness mechanism	Missingness proportion	RFDTI	RFTI
MCAR	10%	2.44%	1.03%
	20%	4.65%	2.33%
	30%	6.98%	4.32%
	40%	9.99%	6.48%
	50%	13.73%	9.16%
MAR	10%	2.47%	1.02%
	20%	4.55%	2.33%
	30%	7.04%	4.15%
	40%	9.94%	6.50%
	50%	13.59%	9.21%
MNAR	10%	1.53%	1.10%
	20%	4.15%	2.99%
	30%	8.09%	5.92%
	40%	11.69%	9.81%
	50%	14.30%	14.14%
MIXED	10%	1.29%	0.86%
	20%	3.65%	2.29%
	30%	7.61%	4.55%
	40%	12.07%	8.00%
	50%	14.83%	12.60%

In general, the remaining missing rate showed an upward trend as the missingness proportion in the original data increased, and it was below or approximate 10% in most conditions considered in this study. Only when the original missing proportion reached 50% under the MCAR and MAR mechanisms, or when the original missing proportion reached 40% under the MNAR or MIXED mechanisms, the remaining missing rate of RFDTI imputed data was about 10% ~ 15%. Therefore, in the subsequent analysis based on the RFDTI imputed data, the remaining missing values after being imputed by RFDTI were temporarily ignored (Hartz et al., 2002; Little and Rubin, 2002; Muthén et al., 2011). In addition, as expected, the remaining missing rates of RFDTI were slightly higher that those of RFTI.

### Classification accuracy of attribute profiles

**Figure 1:**
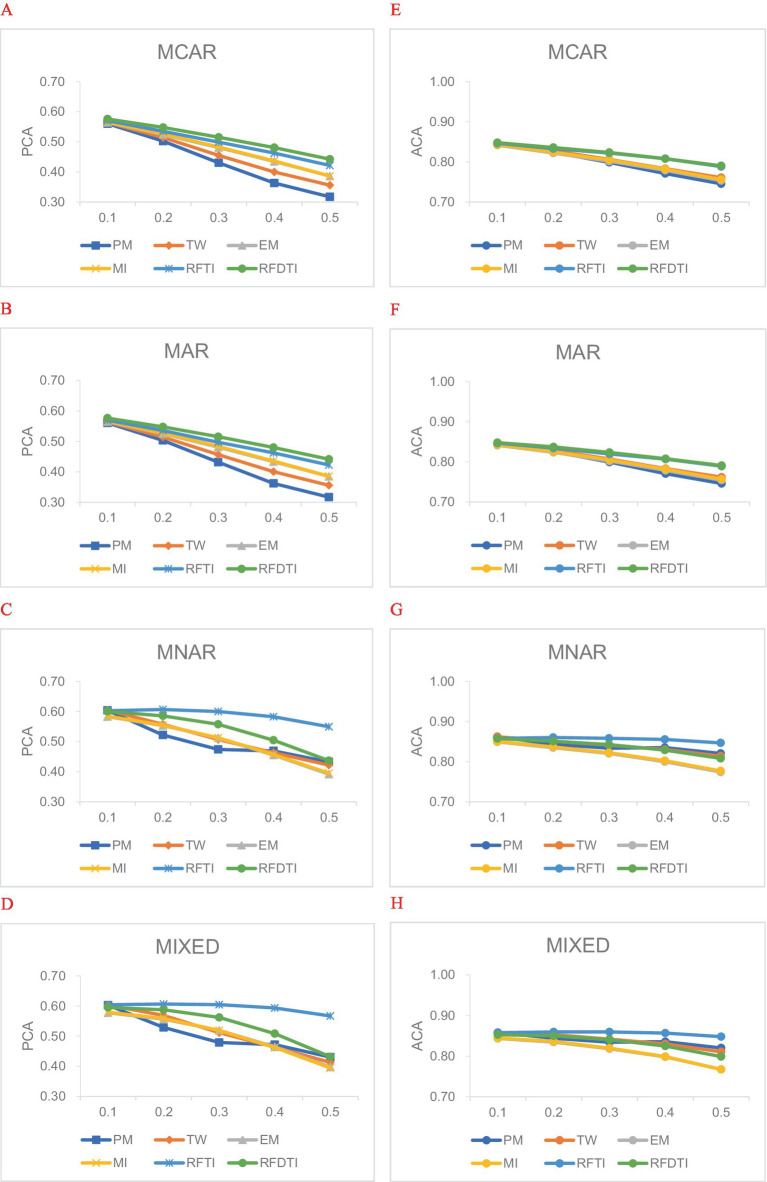
Average attribute classification accuracy under different missing mechanisms and proportions. **(A–D)** PCA values of attribute profiles under four missingness mechanisms. **(E–H)** ACA values of attribute profiles under four missingness mechanisms.

In general, PCA and ACA results had similar trends between methods or the different levels of design factors, while the differences in ACA values were smaller than those in PCA values. Figure 1 shows the average PCA and ACA of the estimated attribute profiles for the six methods under different missingness mechanisms and proportions. According to Figure 1, the higher the missingness proportion, the worse the classification accuracy tends to be.

Then we focused on the comparison between methods. When the missing data was MCAR (Figures 1A,E) or MAR (Figures 1B,F), RFDTI performed very similarly or even slightly better than RFTI, and both outperformed the other four methods, especially when the missing proportion increased. EM and MI performed slightly better in PCA than TW and PM, while these four methods resulted in quite similar ACA results. Under the MNAR (Figures 1C,G) or MIXED (Figures 1D,H) mechanism, RFDTI also performed reasonably well. Specifically, RFDTI performed better than the other four traditional methods (i.e., PM, TW, EM, and MI) in terms of PCA. In addition, based on ACA results, RFDTI performed similarly to PM and TW and better than EM and MI. However, for MNAR or MIXED data, the recovery of attribute patterns based on RFDTI was no better than that from RFTI, and the difference between two methods increased with a larger missingness proportion. This might be related to the fact that the remaining missing rate in RFDTI-imputed data was higher than that of RFTI.

The average PCA and ACA values of each method under different missingness mechanism, missing proportions, number of attributes, or sample sizes are provided in Table A1 of Appendix A. When comparing the results across different missingness mechanisms, we found that the classification accuracy of each method for the MNAR and MIXED data was slightly higher than that for the MAR and MCAR data. This pattern was more apparent for PM, TW, and RFTI. As for the other two design factors (i.e., the number of attributes and the sample size), the classification accuracy for each method tended to decrease when the test measured more attributes, while the sample size had little effect on the classification accuracy for all six methods.

## Empirical study

In this section, we illustrate the application of the proposed RFDTI method using the data collected from a cognitive diagnosis assessment designed for Chinese seventh-grade students. Missing data were MNAR by design, which will be explained in detail in the subsection *Test administration*. Students’ academic achievements in Chinese and mathematics in the final examination and their attribute patterns estimated from the complete data in a parallel test were used as the criteria to evaluate the performance of RFDTI.

### Instrument

We adopted a cognitive aptitude test for seventh-grade students developed by the psychometric research center of Beijing Normal University. It contains two parallel test forms (A and B) with identical test length and structure (i.e., Q matrix). Each test form has 50 items and measures five attributes, including verbal reasoning, analogical reasoning, symbolic operation, matrix reasoning, and spatial reasoning. In each form, each item measures only one attribute, and each attribute is measured by 10 items (see Table B1 of Appendix B). The instruments and assessment procedures were reviewed and approved by the research committee of Beijing Normal University. The school teachers, students, and their parents had a clear understanding of this project and how data was collected. Parents of all student participants approved and signed informed consent forms.

Before using the two forms of the cognitive aptitude test, we performed a prior analysis to examine their instrument quality. We collected response data from 181 and 186 seventh-grade students from Dalian City, Liaoning Province on test forms A and B, respectively. Then, under the classical test theory (CTT) framework, we calculated the difficulty and discrimination of each item, the difficulty and reliability of each attribute and the entire test (see Appendix B). In general, the difficulty of most items was between 0.3 and 0.7 and the discrimination was between 0.3 and 0.5. The test difficulty of the two forms was 0.421 and 0.471, respectively, and their test reliability was 0.870 and 0.899, respectively.

According to the item difficulty, test form A was divided into two subtests with identical length and structure. The easier subtest A1 was composed of the 5 easiest items for each of the five attributes, totaling 25 items. Subtest A2, the more difficult one, consisted of the remaining 5 items for each attribute.

### Test administration

The test administration involved two phases. In the first phase, each student was required to complete test form B within 60 min. The responses were then analyzed using the DINA model to estimate the students’ attribute mastery patterns, which were transformed into attribute mastery scores (i.e., the number of attributes mastered) ranging from 0 to 5. Afterward, all the students were divided into two groups, including a low-level group with attribute mastery scores from 0 to 2, and a high-level group with attribute mastery scores from 3 to 5.

The second phase was conducted two weeks later. The low-level group and the high-level group were administered the easy subtest A1 and the difficult subtest A2, respectively, within 30 min. In this case, the missing responses of the low-level group on subtest A2 and the high-level group on subtest A1 could be regarded as MNAR.

### Sample

A total of 610 seventh-grade students from a junior middle school in Dalian City, Liaoning Province in China participated in the first phase of the test (completing test form B), of which 52.78% were boys and 47.22% were girls. Only 599 of them participated in the second phase (completing test form A1 or A2), including 271 in the low-level group and 328 in the high-level group. Therefore, the sample used for analysis in this study was 599 students, each of whom responded to just half of the items on test form A.

### Analysis

Data analysis consists of two stages: (1) dealing with missing data using different methods and estimating students’ attribute mastery patterns based on responses of subtests A1 and A2; and (2) evaluating the performance of those methods.

In the first stage, we used six methods, i.e., PM, TW, EM, MI, RFTI, and RFDTI, to impute missing data on test form A, respectively, and fit the DINA model to each of the six imputed datasets to obtain a set of estimates of students’ attribute patterns. In order to improve the results of different imputation methods, we added the complete data of 181 students on test form A into the current response data, which were collected for quality analysis of the test instrument (see subsection *Instrument*). Therefore, all the available response data of 181 + 599 = 780 students on Test A were used and the missingness proportion was 38.40% in this stage.

In the second stage, the performance of different imputation methods was evaluated through two external criteria, i.e., attribute patterns estimated from response data on test B and academic achievements, based on the sample of 599 students.

First, we calculated the consistency between the classifications estimated based on the response data on test form B and the data on test A dealt with by each imputation method. Specifically, the DINA model was fitted to the 599 students’ responses to test B, estimating their attribute patterns. These estimates were used as criteria to evaluate the imputation accuracy of the six missing data handling methods. PCA and ACA were still used as the evaluation criteria, while the true attribute patterns in Equations 8, 9 were replaced with the estimated attribute patterns based on test form B. Therefore, PCA and ACA measured the consistency between the classifications estimated based on the data of the two test forms. The higher the consistency, the better the performance of the imputation method.

Next, we calculated the correlation between the estimated attribute mastery scores and the academic achievements in Chinese and mathematics, and then compared these correlations based on test forms A and B. Specifically, for test form A, we calculated the correlations between the academic achievements and attribute mastery scores obtained by using different missing data handling methods, so each method had a corresponding correlation value. Then, the correlations between the attribute mastery scores from test form B and the academic achievements were taken as the comparison standards. A smaller difference between the correlation of a missing data handling method and that coefficient based on test form B indicated better performance of this method.

Note that, as in the simulation study, MI results were pooled by averaging the corresponding measures of the estimation (i.e., PCA, ACA, or the correlation between the estimated attribute mastery scores and external criteria) across all imputed datasets.

### Results

Table 3 shows the PCA and ACA of each missing data handling method for the whole sample, as well as the two groups with different ability levels. In this MNAR design, RFTI always resulted in highest values of PCA and ACA among the methods, above 0.9, both for the whole sample or for subgroups. The proposed RFDTI could provide similar results as RFTI, of which the PCA and ACA values were close to or above 0.90. Among the remaining four traditional methods, TW performed relatively better and its results were similar between the two ability level groups, while PM performed the worst for the whole sample (both PCA and ACA were lower than 0.6) and quite differentially between two groups. The PCA of PM was even below 0.4 for the high-level group, but exceeded 0.8 for the low-level group.

**Table 3:** PCA and ACA of six missing data handling methods.

Method	PCA			ACA		
	Whole sample	Low-level group	High-level group	Whole sample	Low-level group	High-level group
PM	0.576	0.815	0.378	0.581	0.865	0.458
TW	0.788	0.775	0.799	0.810	0.825	0.836
EM	0.701	0.749	0.662	0.723	0.796	0.701
MI	0.650	0.715	0.596	0.683	0.751	0.642
RFTI	0.920	0.915	0.924	0.941	0.931	0.946
RFDTI	0.901	0.899	0.905	0.934	0.928	0.932

Table 4 presents the correlation coefficients between the attribute mastery scores based on two test forms and academic achievements in Chinese and mathematics. The pattern of results among six imputation methods was consistent for the two subjects. The estimated attribute mastery scores after using RFDTI or RFTI to deal with missing data were the most strongly correlated with academic achievements, and these correlations were the closest to those based on the complete responses from test B. MI also performed well, while EM was the worst.

**Table 4:** Correlation between attribute mastery scores and academic achievements.

Test form	Method	Chinese	Mathematics
A	PM	0.271	0.318
	TW	0.371	0.416
	EM	0.253	0.296
	MI	0.455	0.511
	RFTI	0.484	0.530
	RFDTI	0.494	0.523
B	/	0.544	0.613

## Discussion

In this study, we improved the Random Forest Threshold Imputation method proposed by You et al. (2023), which is designed for handling missing data in the implementation of CDMs and demonstrates superiority for MNAR and MIXED data and a large missingness proportion. Specifically, motivated by the fixed lower threshold in RFTI and related uncertainty of imputation, in this study, we adapted the RFTI method by setting both dynamic upper and lower thresholds to increase the imputation accuracy. For ease of application, we also developed an R package *missForestCDA* for the RFDTI method.

Based on the machine learning algorithm, RFDTI is a nonparametric method, and it relies much less on the assumptions of the distribution or the missingness mechanism of the data compared with traditional methods such as EM and MI. Results of the current simulation and empirical studies also demonstrate the effectiveness of the RFDTI method from the perspective of attribute pattern classification. The attribute profile estimations for RFDTI were consistently more accurate than the four traditional methods (PM, TW, EM, MI), even when the missingness proportion was high (>30%). The performance of RFDTI for the empirical MNAR data was also better than the four methods and much closer to the results based on complete data.

However, RFDTI did not show obvious advantages over RFTI. In the simulation, for MCAR or MAR data, RFDTI slightly outperformed RFTI. However, for MIXED and MNAR data, the situation was reversed. The differences between the two methods can be negligible in the case of a small percentage of missingness, but became larger with a higher missingness proportion. This may be related to the percentage of remaining missing data after imputation. When the missing percentage of the original data is higher, the remaining missing percentage after imputation will also be relatively higher, while this part of missing values will not be treated, but just ignored. RFDTI would produce a higher missing percentage than RFTI. According to Table 2, in the case of an original missing percentage of 50%, the remaining missing percentage after imputation using RFDTI could approach 15%, while it is lower in RFTI. Under the MNAR and MIXED missing mechanism, the requirement for the percentage of missing data that can be negligible may be lower. So the accuracy of RFDTI is instead lower than RFTI with a large missing proportion for MNAR or MIXED data.

The findings of this study also indicate the need to pay special attention to the treatment of missing data in CDM applications, which is also one of the starting points of the current study. In the current simulation, the estimation of attribute profiles for each method was better under the MIXED and MNAR mechanisms than under the MAR and MCAR mechanisms. For the traditional missing data handling methods, this finding is inconsistent with previous research results in the IRT context (e.g., Finch, 2008; Wolkowitz and Skorupski, 2013). The reason is likely that the person parameters to be estimated in CDMs are binary variables (i.e., the classification of attribute mastery status), rather than continuous variables (such as latent ability) as in IRT models. Research has found that the performance of missing data handling methods is related to the missingness mechanism and the relationship relies on the specific research contexts, including the analysis model and data type (categorical or continuous) (Dai, 2017; Newman, 2003; Poleto et al., 2011; Song et al., 2022; Zhuchkova and Rotmistrov, 2022; Fu et al., 2025; Qin et al., 2024). Accordingly, it is conceivable that the impact of the missing data mechanism on the traditional methods may differ between CDM and IRT. On the other hand, the good performance of the RFDTI method may be related to its greater use of individual response patterns that may provide additional useful information under nonrandom missingness mechanisms. Therefore, the proposed method and its comparison with traditional methods in this study provide users with more choices of missing data handling methods in CDM applications and provide a basis for the method selection.

Although the RFDTI method seems very promising, there are some issues for further study. First, in this study, we only focus on the estimation of the attribute mastery status, leaving the item parameter estimation aside temporarily. However, how the item parameter estimation of CDMs will be affected when using RFDTI to deal with missing data needs further investigation. Second, in this study, we only explored the performance of the RFDTI method in the context of the common DINA model. In future research, the RFDTI method can be applied in combination with other CDMs, such as the fusion model (Hartz et al., 2002), the hierarchical DINA model (de la Torre and Douglas, 2004; Yan et al., 2025), and the DINO model, so as to explore its performance in more research contexts. Third, simulation conditions related to cognitive diagnosis assessments should be enriched (e.g., varying test lengths and hierarchical relationships of attributes) to more comprehensively demonstrate the performance of the RFDTI approach.

## Data Availability

The original contributions presented in the study are included in the article/Supplementary material, further inquiries can be directed to the corresponding author.
