# Correction: A random forest dynamic threshold imputation method for handling missing data in cognitive diagnosis assessments

**DOI:** 10.3389/fpsyg.2025.1686437

**Published:** 2025-09-12

**Authors:** Xiaofeng You, Jianqin Yang, Xinai Xu

**Affiliations:** School of Mathematics and Information Science, Nanchang Normal University, Nanchang, China

**Keywords:** missing data, cognitive diagnosis assessment, random forest threshold imputation, machine learning, dynamic thresholds

Affiliation: School of Mathematics and Information Science, Nanchang Normal University, Nanchang, China was omitted for author Xinai Xu. This affiliation has now been added for author [Xinai Xu].

Author Xinai Xu was erroneously assigned to affiliation Department of Educational Psychology, Faculty of Education, East China Normal University, Shanghai, China. This affiliation has now been removed for author [Xinai Xu].

In the published article, there was an error in affiliation, 3. Instead of 3 Faculty of Psychology, Beijing Normal University, Beijing, China there should be no affiliation 3. The corrected affiliation is: School of Mathematics and Information Science, Nanchang Normal University, Nanchang, China.

The original article has been updated.

