# Supplementary material for: A random forest dynamic threshold imputation method for handling missing data in cognitive diagnosis assessments
Source: Front Psychol. 2025 Aug 5;16:1487111. doi: 10.3389/fpsyg.2025.1487111 (PMC12362312; doi:10.3389/fpsyg.2025.1487111)
Supplement: Supplementary file 1 [file Table_1.pdf]

Appendix A

Table A1

Average Attribute Classification Accuracy Under Different Missing Mechanisms, Missing Proportions, Numbers of Attributes (K), and Sample Sizes (N)

	PCA						ACA					
	PM	TW	EM	MI	RFTI	RFDTI	PM	TW	EM	MI	RFTI	RFDTI
Missing Mechanism												
MCAR	0.435	0.458	0.478	0.479	0.498	0.512	0.797	0.805	0.800	0.802	0.820	0.821
MAR	0.435	0.458	0.478	0.479	0.497	0.512	0.796	0.805	0.799	0.802	0.819	0.821
MNAR	0.499	0.510	0.499	0.501	0.588	0.537	0.838	0.839	0.815	0.817	0.856	0.838
MIXED	0.502	0.512	0.503	0.503	0.595	0.537	0.838	0.838	0.812	0.813	0.856	0.833
Missing Proportion												
10%	0.582	0.584	0.572	0.574	0.820	0.587	0.852	0.852	0.843	0.845	0.957	0.851
20%	0.513	0.538	0.540	0.542	0.819	0.567	0.834	0.840	0.828	0.830	0.914	0.843
30%	0.453	0.482	0.498	0.499	0.856	0.538	0.816	0.824	0.810	0.812	0.862	0.832
40%	0.417	0.432	0.447	0.448	0.856	0.493	0.803	0.807	0.789	0.791	0.810	0.817
50%	0.375	0.387	0.390	0.390	0.820	0.438	0.783	0.787	0.761	0.764	0.763	0.797
K												
3	0.776	0.807	0.827	0.828	0.882	0.859	0.916	0.928	0.934	0.934	0.957	0.948
4	0.638	0.662	0.654	0.656	0.728	0.698	0.885	0.892	0.882	0.883	0.914	0.902
5	0.486	0.502	0.497	0.499	0.566	0.537	0.842	0.846	0.827	0.829	0.862	0.850
6	0.370	0.382	0.386	0.387	0.440	0.421	0.796	0.798	0.775	0.778	0.810	0.800
7	0.295	0.304	0.313	0.313	0.356	0.343	0.753	0.754	0.730	0.733	0.763	0.754
8	0.243	0.251	0.261	0.260	0.296	0.290	0.713	0.714	0.692	0.694	0.721	0.716
N												
500	0.467	0.484	0.489	0.488	0.544	0.523	0.817	0.821	0.806	0.807	0.837	0.829
1000	0.468	0.484	0.489	0.491	0.545	0.525	0.817	0.822	0.807	0.809	0.838	0.828
2000	0.469	0.485	0.490	0.493	0.546	0.526	0.818	0.822	0.807	0.810	0.838	0.828

Appendix B

Information about Two Test Forms Used in the Empirical Study

Table B1

Item Difficulty and Discrimination of Two Forms of the Cognitive Aptitude Test

Item number	Measured Attribute	Test form A		Test form B	
		Item difficulty	Item discrimination	Item difficulty	Item discrimination
1	Verbal reasoning	0.696	0.385	0.803	0.406
2	Verbal reasoning	0.547	0.446	0.776	0.435
3	Verbal reasoning	0.740	0.132	0.799	0.353
4	Verbal reasoning	0.696	0.423	0.707	0.399
5	Verbal reasoning	0.575	0.324	0.611	0.417
6	Verbal reasoning	0.547	0.209	0.487	0.250
7	Verbal reasoning	0.436	0.363	0.662	0.289
8	Verbal reasoning	0.508	0.312	0.468	0.360
9	Verbal reasoning	0.376	0.320	0.491	0.373
10	Verbal reasoning	0.403	0.162	0.553	0.318
11	Analogical reasoning	0.453	0.333	0.654	0.273
12	Analogical reasoning	0.608	0.402	0.689	0.367
13	Analogical reasoning	0.541	0.358	0.533	0.357
14	Analogical reasoning	0.635	0.402	0.635	0.361
15	Analogical reasoning	0.580	0.259	0.544	0.359
16	Analogical reasoning	0.564	0.465	0.687	0.500
17	Analogical reasoning	0.436	0.275	0.653	0.357
18	Analogical reasoning	0.591	0.326	0.662	0.299
19	Analogical reasoning	0.602	0.442	0.629	0.393
20	Analogical reasoning	0.597	0.434	0.595	0.452
21	Symbolic operation	0.271	0.354	0.313	0.420
22	Symbolic operation	0.260	0.298	0.324	0.382
23	Symbolic operation	0.282	0.233	0.441	0.425
24	Symbolic operation	0.227	0.295	0.302	0.374

Item number	Measured Attribute	Test form A		Test form B	
		Item difficulty	Item discrimination	Item difficulty	Item discrimination
25	Symbolic operation	0.193	0.357	0.311	0.430
26	Symbolic operation	0.215	0.174	0.189	0.447
27	Symbolic operation	0.238	0.392	0.286	0.386
28	Symbolic operation	0.160	0.460	0.245	0.481
29	Symbolic operation	0.381	0.293	0.324	0.432
30	Symbolic operation	0.326	0.319	0.303	0.318
31	Matrix reasoning	0.409	0.571	0.618	0.608
32	Matrix reasoning	0.558	0.536	0.537	0.622
33	Matrix reasoning	0.564	0.523	0.538	0.600
34	Matrix reasoning	0.453	0.585	0.480	0.602
35	Matrix reasoning	0.392	0.471	0.531	0.633
36	Matrix reasoning	0.381	0.575	0.458	0.611
37	Matrix reasoning	0.387	0.503	0.328	0.530
38	Matrix reasoning	0.260	0.381	0.306	0.440
39	Matrix reasoning	0.343	0.310	0.344	0.431
40	Matrix reasoning	0.260	0.380	0.348	0.452
41	Spatial reasoning	0.381	0.349	0.437	0.438
42	Spatial reasoning	0.403	0.373	0.407	0.378
43	Spatial reasoning	0.459	0.352	0.391	0.332
44	Spatial reasoning	0.337	0.354	0.375	0.331
45	Spatial reasoning	0.354	0.330	0.311	0.308
46	Spatial reasoning	0.309	0.259	0.382	0.333
47	Spatial reasoning	0.315	0.486	0.341	0.462
48	Spatial reasoning	0.210	0.376	0.317	0.384
49	Spatial reasoning	0.320	0.377	0.202	0.300
50	Spatial reasoning	0.260	0.339	0.204	0.267

Note. Item difficulty is measured by the proportion of students who answered the item correctly. Item discrimination is measured by the correlation between the item scores and the total test scores.

Table B2

Difficulty of Each Attribute and the Whole Test

	Test form A	Test form B
Verbal reasoning	0.552	0.636
Analogical reasoning	0.561	0.628
Symbolic operation	0.255	0.304
Matrix reasoning	0.401	0.449
Spatial reasoning	0.335	0.337
The whole test	0.421	0.471

Note. The attribute/test difficulty is calculated as the average of the proportion of correctly answered items of the attribute/test across all students.

Table B3

Reliability of Each Attribute and the Whole Test

	Test form A	Test form B
Verbal reasoning	0.592	0.678
Analogical reasoning	0.668	0.674
Symbolic operation	0.778	0.828
Matrix reasoning	0.811	0.861
Spatial reasoning	0.672	0.723
The whole test	0.870	0.899

Note. The reliability is measured by Cronbach's alpha coefficient for internal consistency.
